# A meta-analysis of the efficacy of fractional CO_2_ laser combined with acupuncture for acne scars

**DOI:** 10.1097/MD.0000000000043391

**Published:** 2025-07-18

**Authors:** Jianwei Fang, Tingting Fang, Ruofei Xu, Mei Huang

**Affiliations:** a Dermatology Department, Longyou County People’s Hospital, Longyou, Zhejiang Province, China; b Orthopedics Department, Longyou County People’s Hospital, Longyou, Zhejiang Province, China; c Surgical Department, Beijing University of Chinese Medicine Shenzhen Hospital (Longgang), Shenzhen, Guangdong Province, China.

**Keywords:** acne scars, acupuncture, efficacy, fractional CO_2_ laser, meta-analysis

## Abstract

**Background:**

To systematically evaluate the efficacy of fractional carbon dioxide (CO_2_) laser combined with acupuncture in acne scars based on the available evidence.

**Methods:**

China National Knowledge Infrastructure, Wanfang Data Knowledge Service Platform, Chinese Biomedical Database, China Science and Technology Journal Database, PubMed, Embase, the Cochrane Library, Web of Science databases were searched for published articles on fractional CO_2_ laser combined with acupuncture for acne scars from their inception to March 22, 2024. Meta-analysis of the articles was performed using Stata/SE 15.0 software after an independent screening of the articles by 2 evaluators, extraction of data and then cross-checking, and quality evaluation of the included articles.

**Results:**

A total of 6 articles, involving 468 patients with acne scars, were included. According to a meta-analysis, fractional CO_2_ laser combined with acupuncture (the treatment group) significantly reduced Echelle d’Evaluation Clinique des Cicatrices d’Acne scores compared to fractional CO_2_ laser alone (the control group), the efficacy was considerably better than that of fractional CO_2_ laser alone (relative risk (RR) = ‐3.02, 95% confidence interval [CI] [‐4.47, ‐1.58], *P* = .000; RR = 1.31, 95% CI [1.14, 1.51], *P* = .000). The 2 groups did not differ significantly in patient satisfaction or adverse effects (persistent erythema and hyperpigmentation) (RR = 1.74, 95% CI [0.69, 4.36], *P* = .238; RR = 1.25, 95% CI [0.51, 3.07], *P* = .627; RR = 0.93, 95% CI [0.59, 1.47], *P* = .760).

**Conclusions:**

Based on the available studies, the efficacy of fractional CO_2_ laser combined with acupuncture for acne scars is significantly better than fractional CO_2_ laser alone, and there is no increase in the occurrence of side effects. However, we still need more rigorously designed, large-scale multicenter randomized controlled trials to verify this result.

## 1. Introduction

Acne is a common chronic inflammatory disease in dermatology, mostly involving hair follicles and sebaceous glands.^[1]^ Postpubertal adolescents are more likely to suffer from it, with a prevalence of up to approximately 80%.^[2]^ Improper treatment or the presence of severe acne can lead to the formation of acne scars.^[3]^ Although it can be treated in various ways, it may affect facial aesthetics and cause severe emotional stress to patients, leading to depression and social disorders.^[4,5]^

There are 3 types of acne scars: atrophic, hyperplastic, and keloid, with atrophic scars being the most common. Atrophic scars can be further classified into icepick, rolling, and boxcar types based on their depth, width, and three-dimensional structural features.^[6]^ Currently, laser is a common means of treating acne scars, and fractional carbon dioxide (CO_2_) laser is a new laser method that has achieved better results in clinical treatment and increasingly become the gold standard for treating acne scars.^[7]^ However, fractional CO_2_ laser is often ineffective for icepick scars.^[8]^ How to increase the efficacy of atrophic scars (especially icepick) is an important research topic.

In recent years, studies have shown that acupuncture yields better results in treating icepick scars, with even more effective outcomes when combined with fractional CO_2_ laser treatment.^[9–11]^ This study reviewed clinical trials on the use of acupuncture combined with fractional CO_2_ laser for acne scar treatment and then performed a meta-analysis to evaluate the safety and efficacy of this combination therapy, providing a scientific basis for clinical practice.

## 2. Data and methods

The meta-analysis was registered on PROSPERO (registration number: CRD42022373814) and was conducted according to the Preferred Items for Systematic Review and Meta-analyze 2020 statement.^[12]^

### 2.1. Criterion of inclusion and exclusion

#### 2.1.1. Study type

Randomized controlled trial.

#### 2.1.2. Study subjects

Patients with acne scars of any race, nationality, gender, age, and duration of treatment.

#### 2.1.3. Interventions

Treatment group: fractional CO_2_ laser combined with acupuncture.

Control group: fractional CO_2_ laser alone.

#### 2.1.4. Outcome indicators

Echelle d’Evaluation Clinique des Cicatrices d’Acne (ECCA) scores, efficacy (significant + effective/total number of people), patient satisfaction, adverse outcomes.

#### 2.1.5. Exclusion criteria

Patients with other causes of scars; repeatedly reported studies; studies with incomplete information and relevant data not available; treatment groups included other interventions in addition to fractional CO_2_ laser and acupuncture; review, case report, conference literature, expert opinion, and animal study.

### 2.2. Literature search

A literature search was conducted on China National Knowledge Infrastructure, Wanfang Data Knowledge Service Platform, Chinese Biomedical Database, China Science and Technology Journal Database, PubMed, Embase, the Cochrane Library, Web of Science, with no language limitation from the date of creation to March 22, 2024. The search terms laser, cicatrix, and acupuncture can be used to expand the scope of the search, and the search was performed with a combination of subject terms and free words. The retrieval strategy in Pubmed is shown in Table 1.

**Table 1:** Retrieval strategy in Pubmed.

Steps	Search terms
#1	(“Lasers”[Mesh]) OR (Pulsed Lasers[Title/Abstract]) OR (Laser, Pulsed[Title/Abstract]) OR (Lasers, Pulsed[Title/Abstract]) OR (Pulsed Laser[Title/Abstract]) OR (Continuous Wave Lasers[Title/Abstract]) OR (Continuous Wave Laser[Title/Abstract]) OR (Laser, Continuous Wave[Title/Abstract]) OR (Lasers, Continuous Wave[Title/Abstract]) OR (Masers[Title/Abstract]) OR (Maser[Title/Abstract]) OR (Laser[Title/Abstract])
#2	(“Acupuncture”[Mesh]) OR (Acupuncture[Title/Abstract]) OR (Pharmacopuncture[Title/Abstract])
#3	(“Cicatrix”[Mesh]) OR (Scar[Title/Abstract]) OR (Scars[Title/Abstract]) OR (Cicatrization[Title/Abstract]) OR (Scarring[Title/Abstract]) OR (Cicatrix[Title/Abstract])
#4	#1 AND #2 AND #3

### 2.3. Literature screening and data extraction

The articles were independently assessed by 2 evaluators, who extracted and cross-checked the data and resolved any disagreements through discussion. Regarding literature screening, duplicates were first eliminated, then irrelevant articles were excluded by reading the titles and abstracts, and included articles were identified by reading the full text ultimately. The data extraction mainly included: the first author, time of publication, study site, basic information of subjects (age, sex, disease duration), number of cases in each group, interventions, and treatment course.

### 2.4. Quality evaluation of included studies

The Cochrane Collaboration’s tool risk of bias tool version 2 was used by 2 investigators to independently evaluate the bias risk of the included studies. Specific components of the evaluation included: the methods used for randomization, concealment of allocations, blinding of subjects and investigators, blinding for outcome assessment, completeness of outcome data, the selective reporting of outcomes, and other sources of bias. When there was disagreement in the evaluation results, the 2 investigators resolved the disagreement through discussion and negotiation.

### 2.5. Statistical analysis

The meta-analysis was conducted in Stata/SE 15.0. For dichotomous outcomes, we calculated relative risk (RR) with 95% confidence interval (CI); for continuous outcomes, standardized mean difference with 95% CI were computed. Heterogeneity in studies was assessed using *I*^2^ values. A fixed-effects model was applied when heterogeneity was low (*I*^2^ ≤ 50%), otherwise a random-effects model was employed. Sensitivity analysis was performed by sequentially removing each study and examining its impact on heterogeneity. Publication bias was assessed using funnel plots and Egger test, with statistical significance defined as *P* < .05.

## 3. Result

### 3.1. Results of literature screening

**Figure 1.**
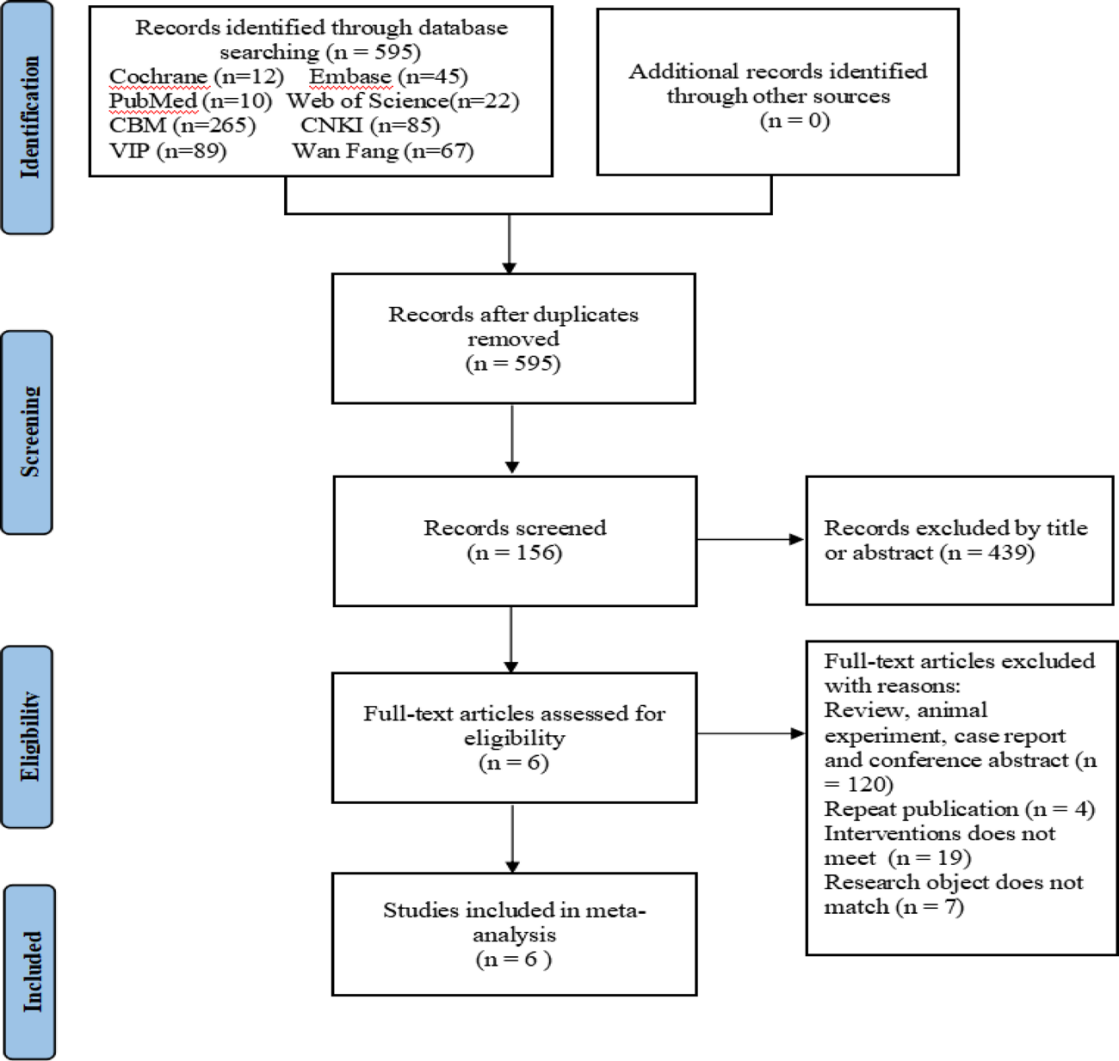
PRISMA flowchart of literature search. PRISMA = The Preferred Items for Systematic Review and Meta-analyze.

The search yielded a total of 595 articles. After screening, 6 articles^[13–18]^ were finally included. A summary of the literature screening process and results can be found in Fig. 1.

### 3.2. Basic characteristics of the included articles

Six articles, which had 468 cases, were included in the study, with half of the sample size in the control group and half in the treatment group. The baseline of the included studies was comparable, and the primary characteristics of the included studies are presented in Table 2.

**Table 2:** Basic characteristics of the included articles.

Study	Study design	Sample size (treatment/control)	Skin types	Intervention duration	Treatment protocol		Outcomes
					Treatment group	Control group	
Zheng XJ 2019	RCT	43/43	Depressed scar (FitzPatrick Ⅲ–Ⅳ)	2 months	Acupuncture + fractional CO_2_ laser	Fractional CO_2_ laser	1. ECCA; 2. adverse outcomes (persistent erythema, hyperpigmentation)
Jiang S 2017	RCT (split face)	20/20	Depressed scar (FitzPatrick Ⅲ–Ⅳ)	6 months	Acupuncture + fractional CO_2_ laser	Fractional CO_2_ laser	1. ECCA; 2. adverse outcomes (persistent erythema, hyperpigmentation); 3. patient satisfaction
Xiao WM 2017	RCT	42/42	Depressed scar (FitzPatrick Ⅲ–Ⅳ)	12 weeks	Acupuncture + fractional CO_2_ laser	Fractional CO_2_ laser	1. ECCA; 2. efficacy; 3. adverse outcomes (hyperpigmentation); 4. patient satisfaction
Xu YR 2021	RCT	50/50	Depressed scar (FitzPatrick Ⅲ–Ⅳ)	12 weeks	Acupuncture + fractional CO_2_ laser	Fractional CO_2_ laser	1. ECCA; 2. efficacy; 3. adverse outcomes (persistent erythema, hyperpigmentation)
Liu GG 2019	RCT	37/37	Depressed scar (FitzPatrick Ⅲ–Ⅳ)	12 weeks	Acupuncture + fractional CO_2_ laser	Fractional CO_2_ laser	1. ECCA; 2. adverse outcomes (persistent erythema, hyperpigmentation)
She XG 2019	RCT	42/42	Depressed scar	6 months	Acupuncture + fractional CO_2_ laser	Fractional CO_2_ laser	1. ECCA; 2. efficacy; 3. adverse outcomes (persistent erythema, hyperpigmentation)

CO_2_ = carbon dioxide, ECCA = Echelle d’Evaluation Clinique des Cicatrices d’Acne, RCT = randomized controlled trial.

### 3.3. Methodological quality assessment of included studies

**Figure 2.**
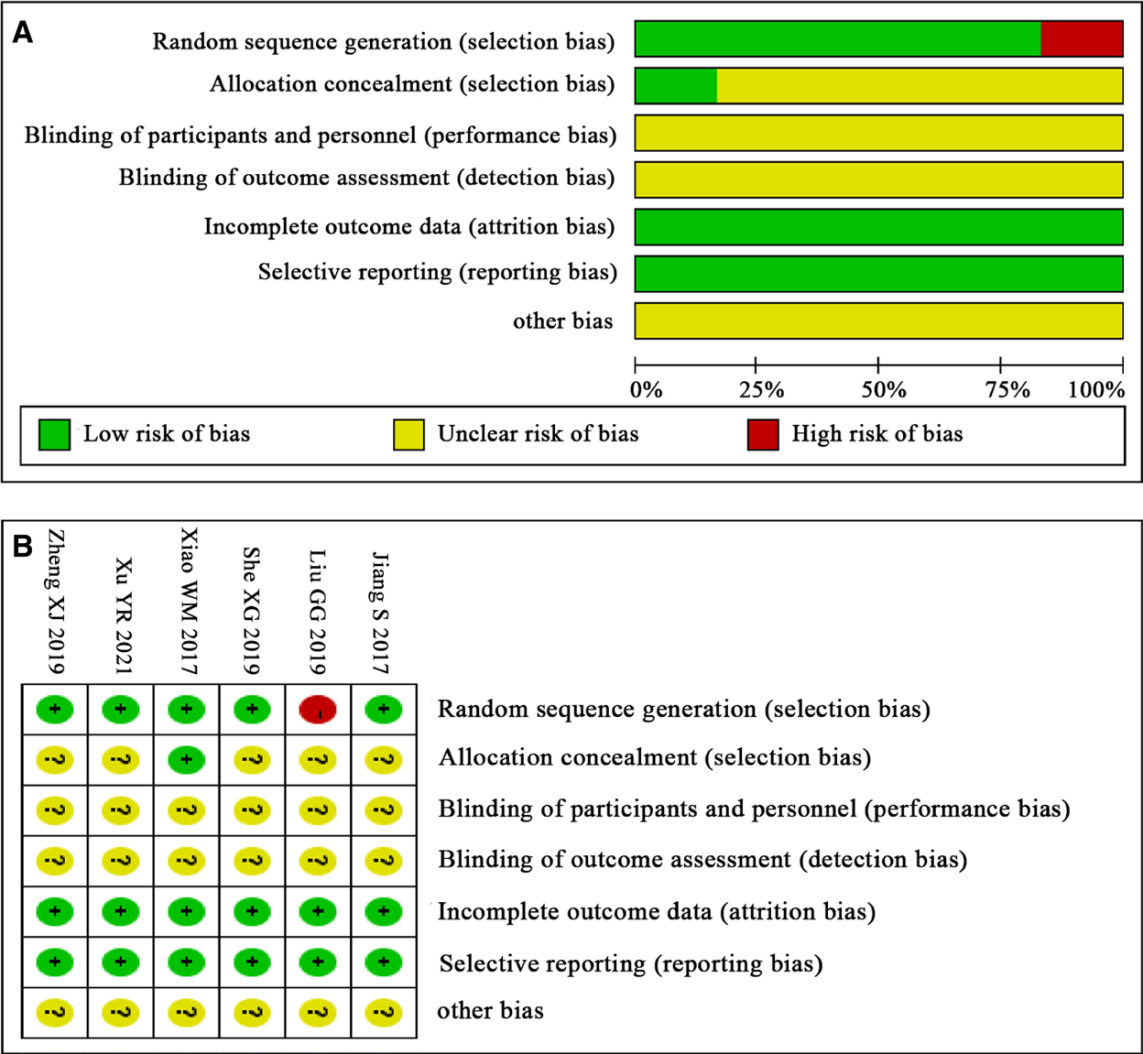
Risks of bias for included studies. (A) Risk of bias graph; (B) risk of bias summary.

Cochrane risk of bias tool version 2 was used to evaluate the risk of bias in the included studies, and the results are illustrated in Fig. 2.

### 3.4. Results of meta-analysis

#### 3.4.1. ECCA scores

**Figure 3.**
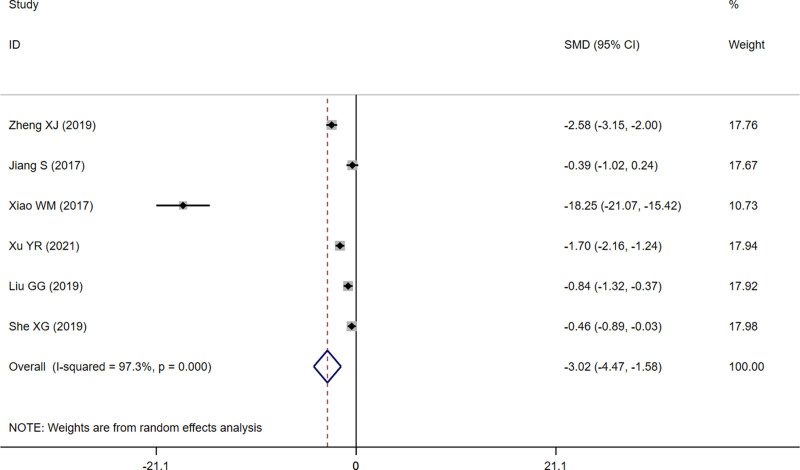
Forest plot of ECCA scores. ECCA = Echelle d’Evaluation Clinique des Cicatrices d’Acne.

Six studies^[13–18]^ reported ECCA scores with a significant heterogeneity among studies (*I*^2^ = 97.3%, *P* = .000). The analysis was conducted using a random-effects model, and the result showed ECCA scores of the treatment group were significantly lower than those of the control group (RR = ‐3.02, 95% CI [‐4.47, ‐1.58], *P* = .000) (Fig. 3).

#### 3.4.2. Efficacy

**Figure 4.**
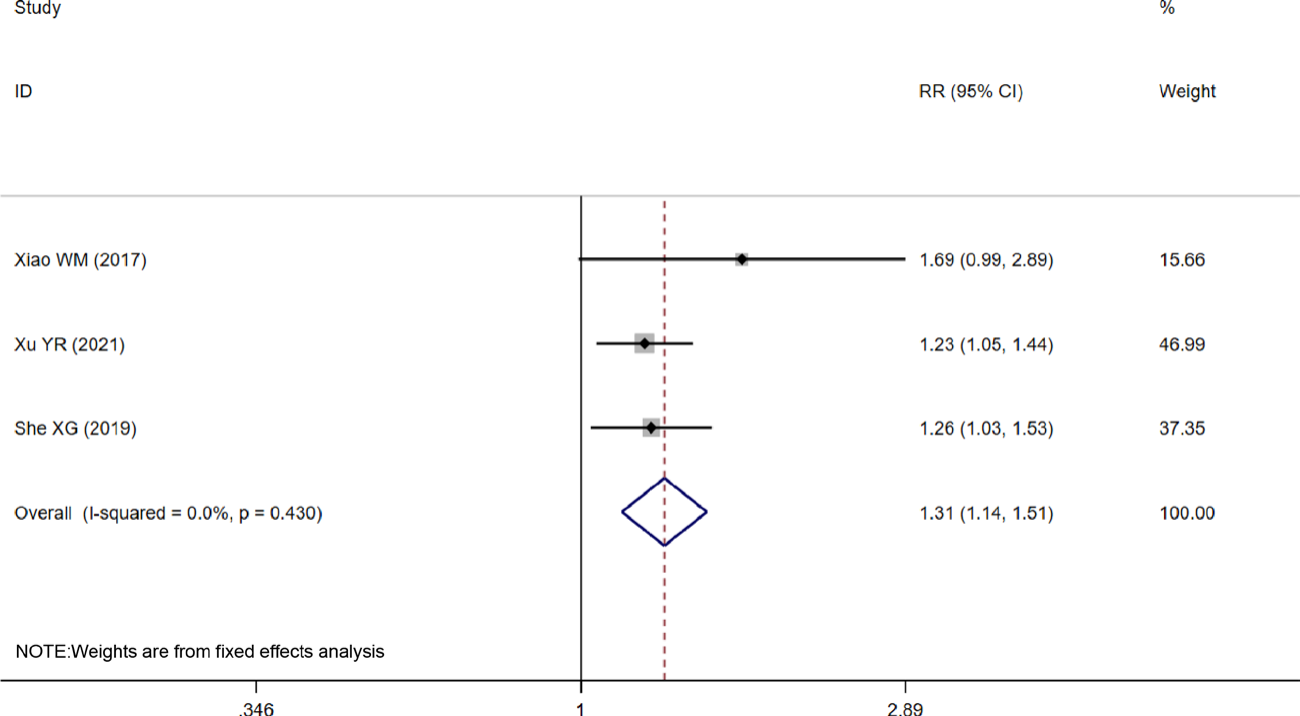
Forest plot of efficacy.

Efficacy was reported in 3 studies,^[13,16,18]^ using a fixed-effects model based on heterogeneous outcomes (*I*^2^ = 0.0%, *P* = .430). The analysis showed significantly better efficacy in the treatment group than in the control group (RR = 1.31, 95% CI [1.14, 1.51], *P* = .000) (Fig. 4).

#### 3.4.3. Patient satisfaction

**Figure 5.**
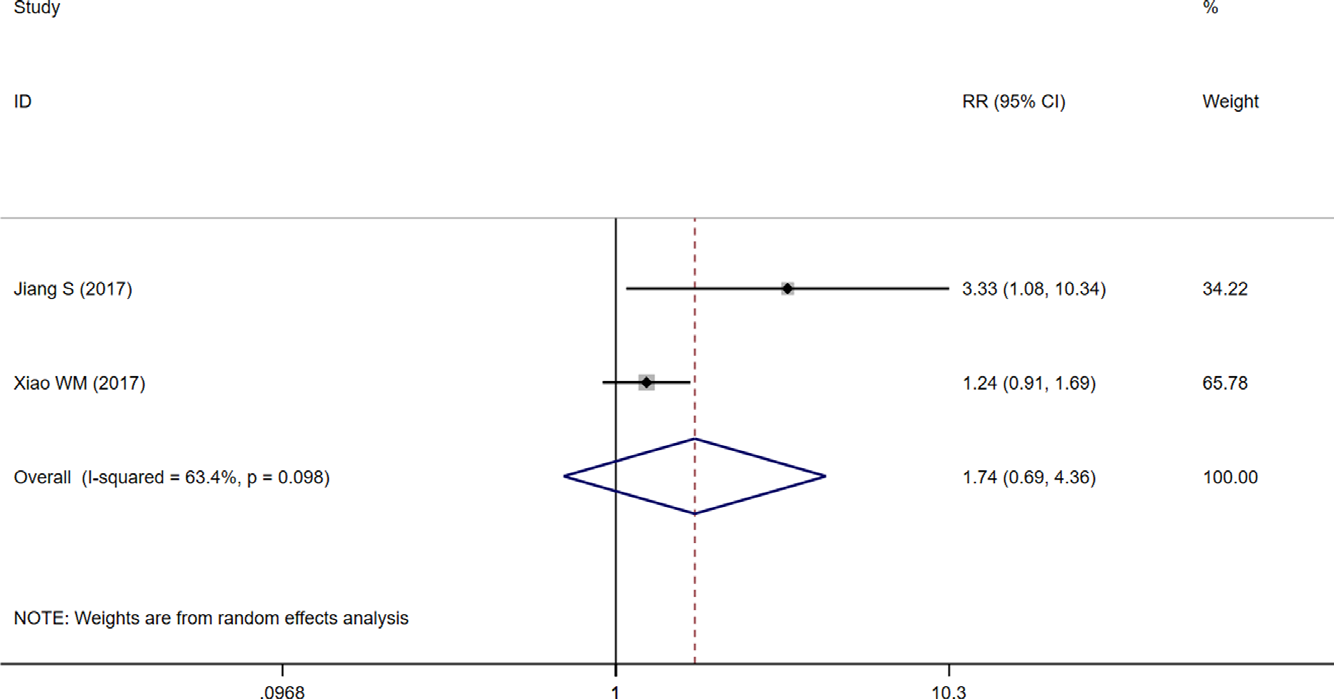
Forest plot of patient satisfaction.

Two studies^[13,14]^ followed up on patient satisfaction, using a random effects model based on heterogeneous results (*I*^2^ = 63.4%, *P* = .098). The analysis showed no significant difference in the number of patients in the treatment group who were satisfied with their treatment compared to the control group (RR = 1.74, 95% CI [0.69, 4.36], *P* = .238) (Fig. 5).

#### 3.4.4. Adverse reaction

**Figure 6.**
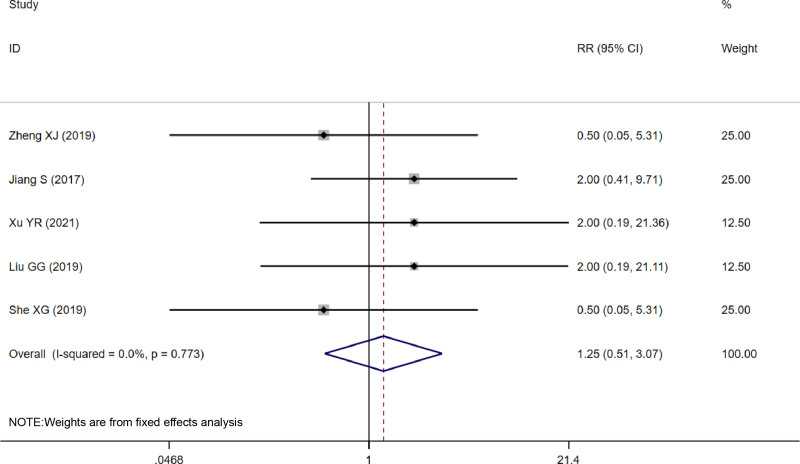
Forest plot of persistent erythema.

**Figure 7.**
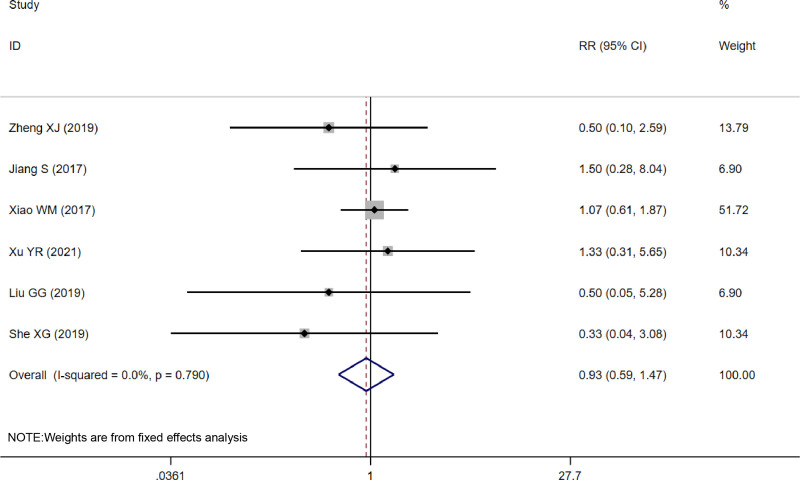
Forest plot of hyperpigmentation.

Six studies^[13–18]^ reported adverse reactions, including persistent erythema, hyperpigmentation, rash, dry skin, and local edema. The most significant adverse reactions were persistent erythema and hyperpigmentation. There was no heterogeneity between studies reporting persistent erythema, and the same was applied to studies reporting hyperpigmentation (*I*^2^ = 0.0%, *P* = .773; *I*^2^ = 0.0%, *P* = .790). The analysis showed that the number of persistent erythema and hyperpigmentation that occurred in the treatment and control groups were similar and not statistically different (RR = 1.25, 95% CI [0.51, 3.07], *P* = .627; RR = 0.93, 95% CI [0.59, 1.47], *P* = .760) (Figs. 6 and 7).

#### 3.4.5. Sensitivity analysis and bias test

**Figure 8.**
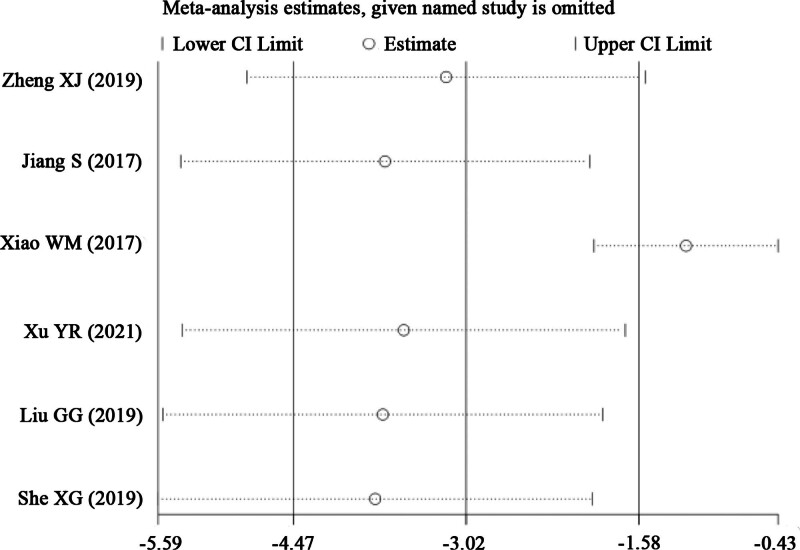
Sensitivity analysis of ECCA score. ECCA = Echelle d’Evaluation Clinique des Cicatrices d’Acne.

**Figure 9.**
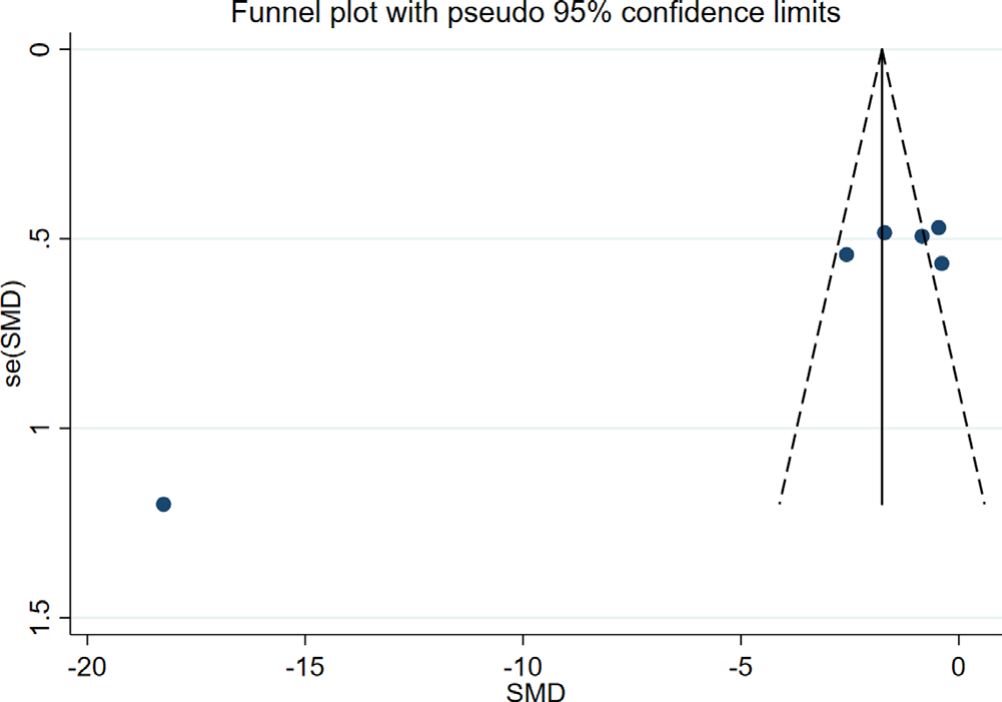
Funnel plot of ECCA score. ECCA = Echelle d’Evaluation Clinique des Cicatrices d’Acne.

Sensitivity analysis and risk of bias assessment were performed on the primary outcome indicator ECCA scores. Excluding the literature one by one, and identified the study by Xiao WM as the primary source of significant heterogeneity (Fig. 8). Comparing this study with other studies, it could be found that its ECCA scores were lower than other studies at baseline and that the posttreatment scores were significantly different from other studies. This may be associated with clinical factors like scar type and individual skin characteristics. Analysis using a random effects model after excluding this study revealed that treatment group ECCA scores remained significantly lower than control group scores, consistent with previous findings (RR = ‐1.19, 95% CI [‐1.96, ‐0.43], *P* = .002). The funnel plot result showed an asymmetrical left-right distribution of scatter, indicating that the studies are at some risk of publication bias (Fig. 9). The quantitative publication bias test using Egger method showed *P* < .05, also suggesting publication bias in the studies, probably due to the inclusion of more low-quality small sample studies with few negative results and all in Chinese literature.

## 4. Discussion

To our knowledge, this is the first systematic evaluation of acne scars treated with acupuncture combined with fractional CO_2_ laser. The subjects in the 6 included papers were all acne depression scars, 5 of which described FitzPatrick types III to IV. The fractional CO_2_ laser is based on the principle of fractional photothermolysis to produce tiny thermal wounds, achieve uniform thermal damage at specific depths within the skin, and create new collagen formation to treat acne scarring.^[19,20]^ In atrophic acne scars, the ice pick type of scar reaches deep into the border between the dermis and the subcutaneous tissue, and there is a fibrous tract pull between the base of the scar and the deeper tissue.^[3,11]^ The fractional CO_2_ laser has limited penetration depth, and for this type of scar, the fractional CO_2_ laser alone often does not achieve better results.^[21]^ Needle fracture therapy, by controlling the depth of needle penetration directly into the dermis and subcutaneous tissue, destroys the abnormal structure of the lesion, loosens the tight fibers in the depressed area, and stimulates blood circulation and collagen regeneration. The combination of fractional CO_2_ laser and acupuncture therapy can play a synergistic role in promoting the continuous synthesis and secretion of dermal collagen, as well as rearranging fibers.^[22,23]^

This meta-analysis evaluates the efficacy and safety of acne scar treatment with acupuncture combined with fractional CO_2_ laser. In terms of efficacy, the treatment group was significantly more effective than the control group. In addition, ECCA is commonly used to assess the severity of acne scars.^[24]^ The study demonstrated significantly lower ECCA scores in the treatment group compared to the control group, indicating that acupuncture combined with fractional CO_2_ laser more effectively improves scar severity in acne scar patients. In terms of safety, adverse reactions including persistent erythema, hyperpigmentation, rash, dry skin, and local edema were observed in both the treatment and control groups. The analysis showed no statistically significant difference in the number of persistent erythema and hyperpigmentation between the treatment and control groups. The analysis of the available included articles showed that acupuncture combined with fractional CO_2_ laser was more effective than fractional CO_2_ laser alone in treating depressed acne scars without increasing the incidence of adverse effects.

This meta-analysis also has some limitations. First, many of the included studies appear to lack sufficient detail on randomization methods and blinding, which may have some impact on the current findings. Second, all studies were conducted in China and most were published in Chinese, which has potential regional bias and the limitation this poses for global generalizability. Third, none of the studies reported whether the blinded grouping of investigators, patients, and outcome measures along with the enrollment assignment concealment scheme, which may result in implementation bias, measurement bias, and selectivity bias, among others. Fourth, certain outcome indicators (e.g., ECCA) involve inherent subjectivity due to inter-researcher variability, leading to widely divergent scoring results and increased study heterogeneity. Fifth, the limited number of included studies (n = 6) and their methodological limitations may reduce the validity of funnel plot analysis for detecting publication bias. The reliability of the results may also be affected, and the efficacy of acupuncture combined with fractional CO_2_ cannot yet be evaluated positively, which is not yet conducive to clinical promotion. The majority of clinicians should analyze and apply the results of this study with caution, taking into account the patient’s specific conditions.

Future rigorously designed, large-scale multicenter randomized controlled trials are needed to establish robust clinical evidence regarding the combined use of acupuncture and fractional CO_2_ laser for acne scar treatment, which will better inform clinical practice.

## Author contributions

**Conceptualization:** Tingting Fang.

**Data curation:** Ruofei Xu, Mei Huang.

**Investigation:** Ruofei Xu.

**Methodology:** Tingting Fang.

**Supervision:** Jianwei Fang.

**Validation:** Jianwei Fang, Mei Huang.

**Writing – original draft:** Tingting Fang, Ruofei Xu.

**Writing – review & editing:** Jianwei Fang, Mei Huang.

## References

[1] Williams HC Dellavalle RP Garner S. Acne vulgaris. Lancet. 2012;379:361–72. 21880356 10.1016/S0140-6736(11)60321-8

[2] Tan JK Bhate K. A global perspective on the epidemiology of acne. Br J Dermatol. 2015;172:3–12. 10.1111/bjd.13462 25597339

[3] Chilicka K Rusztowicz M Szyguła R Nowicka D. Methods for the improvement of acne scars used in dermatology and cosmetology: a review. J Clin Med. 2022;11 :2744. 35628870 10.3390/jcm11102744 PMC9147527

[4] Tan J Chavda R Leclerc M Dréno B. Projective personification approach to the experience of people with acne and acne scarring-expressing the unspoken. JAMA Dermatol. 2022;158:1005–12. 35857307 10.1001/jamadermatol.2022.2742 PMC9301587

[5] Mohamed NE Shabaan SN Raouf AH. Microbotox (Mesobotox) versus microneedling as a new therapeutic modality in the treatment of atrophic post-acne scars. J Cosmet Dermatol. 2022;21 :6734–41. 36169570 10.1111/jocd.15419

[6] Fabbrocini G Annunziata MC D’Arco V. Acne scars: pathogenesis, classification and treatment. Dermatol Res Pract. 2010;2010:893080. 20981308 10.1155/2010/893080 PMC2958495

[7] Aljefri YE Ghaddaf AA Alahmadi RA. Ablative fractional carbon dioxide laser combined with autologous platelet-rich plasma in the treatment of atrophic acne scars: a systematic review and meta-analysis. Dermatol Ther. 2022;35:e15888. 36183145 10.1111/dth.15888

[8] Xu Y Deng Y. Ablative fractional CO_2_ laser for facial atrophic acne scars. Facial Plast Surg. 2018;34:205–19. 29304516 10.1055/s-0037-1606096

[9] Bhargava S Kroumpouzos G Varma K Kumar U. Combination therapy using subcision, needling, and platelet-rich plasma in the management of grade 4 atrophic acne scars: a pilot study. J Cosmet Dermatol. 2019;18:1092–7. 30924301 10.1111/jocd.12935

[10] Alam M Han S Pongprutthipan M. Efficacy of a needling device for the treatment of acne scars: a randomized clinical trial. JAMA Dermatol. 2014;150:844–9. 24919799 10.1001/jamadermatol.2013.8687

[11] Yang Z Jiang S Zhang Y. Self-contrast study of pinprick therapy combined with super pulse fractional CO(2) laser for the treatment of atrophic acne scars. J Cosmet Dermatol. 2021;20:481–90. 32585741 10.1111/jocd.13568

[12] Page MJ Moher D Bossuyt PM. PRISMA 2020 explanation and elaboration: updated guidance and exemplars for reporting systematic reviews. BMJ. 2021;372:n160. 33781993 10.1136/bmj.n160 PMC8005925

[13] Xiao W Chen X. Efficacy of ultrapulse fractional CO_2_ laser combined with acupuncture needling on treatment of facial depressed acne scars. Chin J Aesthet Med. 2017;26:68–71.

[14] Jiang S Zhang Y Chen Y. A self-controlled study of pinprick therapy combined with fractional CO2 laser for atrophic acne scars. Chin J Dermatovenereol. 2017;31:912–5.

[15] Zheng X. Efficacy of fractional CO_2_ laser combined with acupuncture therapy in the treatment of depressed acne scars. Mod Diagn Treat. 2019;30:3072–4.

[16] She X Ma H Luo Y. Analysis of the mechanism of action of fractional laser combined with microneedle therapy for depressed acne scars. World Latest Med Inf. 2019;19:147–50.

[17] Liu G Li H. Clinical efficacy of acupuncture combined with fractional carbon dioxide laser in the treatment of depressed acne scars. Pract Clin J Integr Tradit Chin Western Med. 2019;19:141–3.

[18] Xu Y. The observation of the curative effect of the treatment of acne scar by ultra pulse carbon dioxide lattice laser combined with acupuncture. China Med Device Inf. 2021;27:156–7.

[19] Kar BR Raj C. Fractional CO(2) laser vs fractional CO(2) with topical platelet-rich plasma in the treatment of acne scars: a split-face comparison trial. J Cutan Aesthet Surg. 2017;10:136–44. 29403184 10.4103/JCAS.JCAS_99_17 PMC5782437

[20] Ratanapokasatit Y Sirithanabadeekul P. The efficacy and safety of epidermal growth factor combined with fractional carbon dioxide laser for acne scar treatment: a split-face trial. J Clin Aesthet Dermatol. 2022;15:44–8. PMC9345195 35942017

[21] Jin W Li Z Jin Z Jin C. A novel technique for treating atrophic facial scars in Asians using ultra-pulse CO(2) laser. J Cosmet Dermatol. 2020;19:1099–104. 32073746 10.1111/jocd.13335

[22] Gupta A Kaur M Patra S Khunger N Gupta S. Evidence-based surgical management of post-acne scarring in skin of color. J Cutan Aesthet Surg. 2020;13:124–41. 32792773 10.4103/JCAS.JCAS_154_19 PMC7394107

[23] Zhang Y Chen Y Mei S. Advances in fractional carbon dioxide laser combined with other therapies of atrophic acne scar. Chin J Dermatovenereol. 2022;36:104–7.

[24] Dreno B Khammari A Orain N. ECCA grading scale: an original validated acne scar grading scale for clinical practice in dermatology. Dermatology. 2007;214:46–51. 17191047 10.1159/000096912

